# Medial Mandibulotomies: Is there sufficient space in the midline to allow a mandibulotomy without compromising the dentition?

**DOI:** 10.1186/1916-0216-42-32

**Published:** 2013-05-02

**Authors:** Tulika Shinghal, Eric Bissada, Hon Biu Chan, Robert E Wood, Eshetu G Atenafu, Dale H Brown, Ralph W Gilbert, Patrick J Gullane, Jonathan C Irish, John Waldron, David P Goldstein

**Affiliations:** 1 Department of Otolaryngology-Head and Neck Surgery/Surgical Oncology, Wharton Head and Neck Program, University Health Network, Princess Margaret Hospital, University of Toronto, 610 University Ave 3-952, Toronto, ON M5G 2M9, Canada; 2 Department of Radiation Oncology, Princess Margaret Hospital, University of Toronto, Toronto, Ontario, Canada; 3 Department of Dental Oncology, Ocular and Maxillofacial Prosthetics, Princess Margaret Hospital, University of Toronto, Toronto, Ontario, Canada; 4 Department of Biostatistics, Princess Margaret Hospital, University of Toronto, Toronto, Ontario, Canada

**Keywords:** Mandibulotomy, Complications, Median, Paramedian, Dental, Medial

## Abstract

**Objectives:**

The objective of this study was to determine the frequency of complications in median and paramedian mandibulotomies. In addition, the interdental space in the median and paramedian region was calculated.

**Study design:**

Retrospective study.

**Setting:**

Tertiary care center.

**Methods:**

A retrospective chart review was performed for all cases where a mandibulotomy was performed from 2002 to 2010. 117 charts (61 paramedian and 56 median) were identified. We included data on complications, which fell in the following 2 categories: plate and dental complications. For our second objective, we evaluated 40 different patients with base of tongue or tonsillar cancer treated with intensity modulated radiation therapy (IMRT). The interdental space between the lateral incisors and the canines was electronically calculated on the digital Panorex images.

**Main outcome measures:**

Dental and plate complications were evaluated. We also assessed interdental space.

**Results:**

Patient characteristics were not significantly different. The median group had significantly more dental complications (p=0.0375, RD=0.19 and 95% CI (0.0139-0.3661)). The paramedian group had significantly more plate complications (p=0.0375, RD=0.082 and 95% CI (0.0131-0.1508). The distance between the central incisors was significantly less than the distance between the lateral incisors and canines both at the crestal and apical levels (p=0.0086 and p<0.001).

**Conclusions:**

There are significantly more dental complications in the median approach. There were significantly more plate complications in the paramedian group. In addition, there is significantly less space in the between the median region as compared to the paramedian region. This is the first study that documents the advantage of the paramedian approach for dental complications.

## Introduction

Mandibulotomy is a common technique used to improve exposure for the resection of tumours of the oral cavity, oropharynx and occasionally the parapharyngeal space. It allows for excellent exposure and precise excision of large tumours of these regions [1]. Roux first described mandibulotomy or mandibular swing in 1836 [2]. In 1959, Dubner and Spiro developed a technique with paralingual extension, which is the origin of the modern mandibulotomy.

There are various modifications that can be used in the mandibulotomy procedure. It can be performed anterior (medial or paramedian mandibulotomy) or posterior (lateral mandibulotomy) to the mental foramen. The latter is seldom used due to injury to the inferior alveolar neurovascular canal.

The initial description of the medial or midline mandibulotomy was an osteotomy between the central incisors [3]. Later, in 1991, Spiro and Dubner advocated a paramedian approach between the lateral incisor and the canine, rather than a median approach [4]. The paramedian approach conserved the anterior belly of the digastric, the genioglossus and geniohyoid muscles. They believed this approach prevented muscle necrosis and dead space in the submental region, which would lead to enhanced healing [4,5]. In addition, it is believed that the median approach may result in a delay in swallowing function due to the disruption of the genioglossus and the geniohyoid muscles [6].

Potential complications of mandibulotomy in general, whether it is median or paramedian, include mal-union, non-union, malocclusion, dental complications and potentially osteoradionecrosis if the mandibulotomy is included in the radiation fields [7]. The reported rates of complications related to mandibulotomy range from 10-47% [4,6,8,9]. One of the complications not well described with mandibulotomy techniques are dental complications. Mandibulotomy sites have previously been studied on a limited basis in terms of anatomic and radiographic space availability [5,10]. The primary objective of this study was to determine if there is a difference in the frequency of dental related complications between the median and paramedian mandibulotomies. The secondary objective was to evaluate the interdental space between the teeth at the median mandibulotomy and paramedian mandibulotomy sites.

## Methods

**Figure 1:**
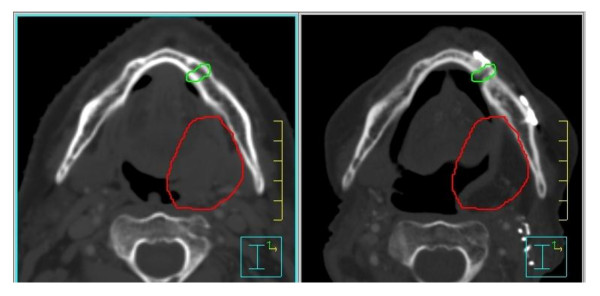
Pre operative treatment distribution planning (left) and fused postoperative CT to assess radiotherapy dose (right).

**Figure 2:**
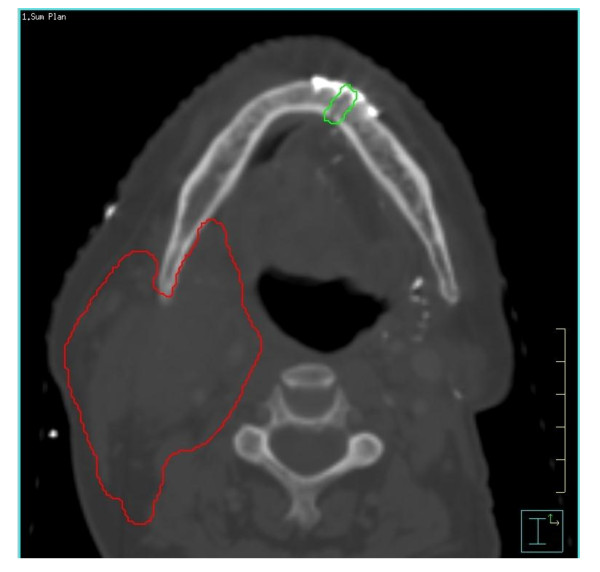
Post-operative treatment distribution with mandibulotomy site visualized.

A retrospective chart review was performed for all patients who underwent a mandibulotomy for resection of oral cavity and oropharynx cancer from 2002 to 2010 at the Princess Margaret Cancer Centre (PMCC), Toronto, Canada. Institutional Ethics Review Board approval was obtained. Patients were included if they underwent a mandibulotomy, either median or paramedian, as part of their primary surgical management for oral cavity cancer or for salvage surgery of recurrent oropharynx or oral cavity cancer. Patients with incomplete charts or who were lost to follow-up were excluded. Patients were also excluded if they did not have dentition around the osteotomy site or if they underwent a mandibulectomy. Eligible patients were identified from a prospective head and neck surgical registry that included patients from 2002 to present and a retrospective oral cavity database, which included all patients undergoing management of oral cavity cancer between 1994 and 2004. A comprehensive review of all medical and available dental charts (dental charts were not available for all the patients) was performed. The data collected included socio-demographics, comorbidities, stage and location of tumour, prior dental assessment, previous treatment (surgery, radiation or chemotherapy), location of the osteotomy, whether the reconstruction plate was contoured before or after the mandibulotomy (pre or post plating), as well as complications. Radiation data collected included method of delivery (conventional vs. intensity modulated radiation therapy (IMRT)), the prescribed dose and fractionation as well as the mean dose to the mandibulotomy site. The dose at the osteotomy site was measured on the original treatment after contouring the mandibulotomy site over a width of 5 mm. For the post radiotherapy (RT) mandibulotomies, we fused postoperative computerized tomography (CT) scans with the original treatment distribution, in order to estimate the preoperative dose received by the mandibulotomy site (Figures 1 and 2).

There is surgeon variability at the PMCC in terms of preference for median or paramedian mandibulotomy. Although there may be some selection of the mandibulotomy location based on tumor and patient characteristics, in the majority of circumstances the site of osteotomy is dependent on the surgeon’s philosophy of mandibulotomy. The mandibulotomy is performed with either a reciprocating or oscillating saw depending on the surgeon’s preference. It is done in a vertical fashion without a step; typically teeth are not extracted for the osteotomy. The mandible is stabilized using a 2.3 non-compression bicortical plate with 3 holes on either side of the osteotomy. This non-compression plate is typically placed towards the lower aspect of the mandible, low enough to avoid the dental roots. In addition, a tension band mini plate with monocortical screws is used along the upper border of the mandible across the osteotomy site. Care is taken to avoid the dental roots with the mandibulotomy.

Complications were divided into 2 categories: dental and plate complications. There were five dental complications taken into account: sensitivity (as described by the patient or demonstrated on a formal dental assessment), periodontal damage (mobility, gingival retraction or formal dental assessment), loss of dentition or extraction, impingement of the osteotomy site on the periodontal ligament or dental root (visible on imaging either a CT scan or a Panorex) and finally need for endodontic treatments. Dental complications were only taken into account if they were not present before surgery based on either new complaints in the medical charts or new abnormalities on formal dental assessments. In addition, these complications had to involve the dentition adjacent to the osteotomy site. Plate complications included plate exposure or plate fracture. We also looked at the incidence of ORN in both groups.

For our secondary objective, a convenience sample of 40 patients with base of tongue or tonsillar cancer treated with IMRT was eligible for inclusion. This was a separate group of sequential patients that were chosen because they all had pre-RT digital Panorexes available. These patients were identified and chosen from a dental database from 2009 onwards. Patients all had a pre-treatment dental evaluation, including a Panorex dental film of the mandible. They all had intact anterior dentition between the first premolars. Patients who had major dental work, missing or malpositioned teeth were excluded. The interdental space was electronically calculated on the digital Panorexes between the central incisors as well as between the lateral incisors and canines. The space was calculated at two levels: at the tooth’s apex and the crest. The dental measurements were performed by one of the authors (TS). The interdental distance was measured at the level of the crestal and apical bones at points, which are anatomically recognized as crestal and apical levels in the dentition. The measurements were performed with digital rulers to prevent miscalculations. These patients were separate from the cohort of patients undergoing mandibulotomy. Hence, the author was not blinded. The measurements were done using the measure tool on Adobe Photoshop and correcting for the approximate 8% magnification on images. Two measurements were taken and the average was used as the final for each the crestal as well as the apical distance.

### Statistical analysis

Patient demographics and treatment related outcomes were reported using descriptive statistics. Categorical variables such as patient’s gender, diabetes, immunosuppression, prior radiation, prior chemotherapy, complications and post-operative RT were summarized with counts and percentages. Continuous variables such as age, hospital stay and interdental distances between the different teeth were summarized with means and standard deviation, or median and range as necessary.

Student’s t-test /Wilcoxon rank-sum test (as appropriate) was used to compare continuous variables between the median mandibulotomy and paramedian mandibulotomy groups. Chi-square test / Fisher’s exact test (as appropriate) was also used to compare categorical variables between the median and paramedian mandibulotomy groups.

For the secondary analysis, mixed model regression analysis was used to compare the interdental distances between the different teeth. All P-values were 2-sided and for the statistical analyses, p < 0.05 was considered to indicate a significantly different result and for multiple testing adjustment was made following the method of Benjamini, Hochberg and Yekutieli to control the false discovery rate [11,12]. Data analysis was performed using Statistical Analysis Software (SAS) Version 9.2.

## Results

There were 117 patients eligible for inclusion, of which 61 underwent the paramedian approach and 56 had a median approach. A summary of the socio-demographics and treatment related variables for the entire cohort, as well as for each group is presented in Table 1. There were no differences between the two mandibulotomy groups, in terms of mean age, gender, history of diabetes, immunosuppression, previous chemotherapy and length of hospital stay. Twenty-three patients had a pre-operative dental assessment, of which none had any dental complaints in the region of the osteotomy site. There were 59 cases of post-plating and 58 cases of pre-plating. Post plating was significantly more common in the paramedian group (42) vs. the median group (17) (p<0.0001). In terms of radiotherapy, 68 patients received postoperative RT and 28 patients received preoperative RT. There was no statistically significant difference in the need for post-operative RT with 34 patients in each group. However, 21 patients in the paramedian group had undergone pre-operative RT vs. 7 in the median group (p=0.006). Of the 96 patients who underwent RT, the mean dose for the median mandibulotomy site was 5337.39 cGY (+/-315.15; max: 5851.64; min: 4019.41) and the paramedian site received 5344.42 cGY (+/- 255.70; max 5853.21; min: 4486.86). There was no statistically significant difference between the mean, maximal or minimal doses received by the two groups (p=0.986, p=0.889 and p=0.313 respectively). The median time from surgery to post-operative RT was 68 days.

**Table 1:** Demographics and group characteristics

	**Paramedian n= 61**	**Median n = 56**	**p-value**
Age (years) mean (SD)	62.13 (12.31)	63.98(13.85)	0.45
Sex (F/M)	19/42	19/37	0.70
Diabetes n (%)	4 (6.56%)	4 (7.14%)	0.99
Immunosuppression n (%)	2 (3.28%)	6 (10.71%)	0.15
Pre-operative Radiotherapy n (%)	20 (32.79%)	7 (12.50%)	0.009
Prior Chemotherapy n (%)	4 (6.56%)	0 (0%)	0.12
Mean hospital stay (days) mean (SD)	25.84 (22.98)	22.21 (14.61)	0.32
T1 n (%)	5 (8.20%)	3 (5.36%)	0.49
T2 n (%)	25 (40.98%)	21 (37.50%)	0.75
T3 n (%)	22 (36.07%)	14 (25.00%)	0.21
T4 n (%)	8 (13.11%)	17 (30.36%)	0.02
N0 n (%)	24 (39.34%)	21 (37.50%)	0.89
N1 n (%)	14 (22.95%)	8 (14.29%)	0.24
N2 n (%)	24 (39.34%)	27 (48.21%)	0.39
N3	0	0	N/A
M0	61	56	N/A
Post-operative Radiotherapy n (%)	34 (55.73%)	34 (60.71%)	0.52

### Complications

Twenty-nine patients had dental complications in the median group (52%) as compared to 20 patients in the paramedian (32%) group (p=0.038, RD=0.19 and 95% CI (0.0139-0.3661)). The distribution of dental complications is listed in Table 2. There were 24 cases of dental sensitivity, 13 cases of periodontal damage, 20 cases of tooth loss, 10 cases of impingement of the osteotomy site and finally 3 patients needed endodontic treatment. Among the dental complications, there were 17 patients who experienced dental sensitivity in the median group vs. 7 in the paramedian group (p=0.01). 10 patients in the median group had periodontal damage vs. 3 in the paramedian group (p=0.04). Mean and median time from surgery to sensitivity was 70 days and 56 days, respectively. There were 5 cases (4.3%) of plate-related complications, all of which occurred in the paramedian group (p=0.04, RD=0.082 and 95% CI (0.0131-0.1508)) (Table 3). Plate exposure was more frequent with post-plating (p=0.03). A multivariable analysis (MVA) was performed for dental complications. Pre vs. post- operative RT and surgery (median vs. paramedian) variables were entered into the model. The result shows that the odds ratio of having dental complication in the pre-operative RT is 0.582 (95% CI 0.270-1.257) to that of post-operative RT. Of note, pre- vs. post-operative RT was not associated with dental complications in the MVA (p=0.1682). There were not enough events in the plate complication to perform a MVA.

**Table 2:** Dental complications in the two groups

	**Sensitivity**	**Periodontal**	**Loss of teeth**	**Impingement**	**Endodontic treatment**
Median	17	10	9	7	2
Paramedian	7	3	11	3	1

**Table 3:** Comparison of plate and dental complications

	**Paramedian (n=61)**	**Median (n=56)**	**Risk diff**	**95% CI for risk difference**	**p-value**	**Adjusted p-value**
Plate complication	5 (8.20%)	0 (0%)	0.082	0.0131-0.1508	0.0285	**0.0375**
Dental complication	20 (32.79%)	29 (51.79%)	0.19	0.0139-0.3661	0.0375	**0.0375**

Although not a primary outcome in the study, the overall incidence rate of osteoradionecrosis (ORN) was 6.84% (8 out of 117). Although limited by patient numbers there was no statistically significant difference in ORN between the median and paramedian groups (p=0.06) or whether patients underwent pre-operative or post-operative RT (p=0.24).

### Evaluation of interdental distances

**Figure 3:**
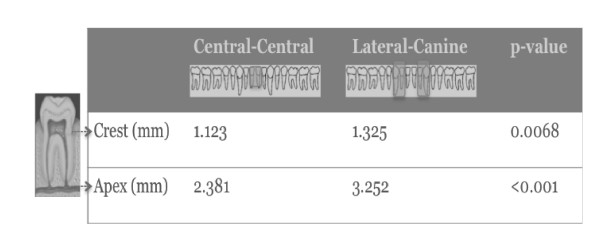
Interdental space was significantly more between the laterals and the canines as compared to the central incisors.

The sample of patients used for calculating interdental space had similar demographics as the mandibulotomy group in terms of age and sex (p>0.05). The mean crestal distance between the lateral incisor and the canine (1.33 mm) was significantly more greater than the distance between the central incisors (1.12 mm) (p=0.0068). Similarly, the mean apical distance between the lateral and the canine (3.25 mm) was more than the distance between the central incisors (2.38 mm) (p<0.0001) (Figure 3).

## Discussion

The decision to chose either the median or paramedian approach is based on the location of the tumour, history of prior RT, as well as the surgeon’s preference. Proponents of the paramedian approach suggest that by preserving the anterior belly of digastric, geniohyoid and genioglossus, patients have superior swallowing function [6,13]. In addition, with less dead space and necrosis, there is a theoretical lower risk infection [4]. The paramedian approach leads to a better-preserved vascular supply due to the maintenance of the muscular contribution in the central region. Lastly, due to the lack of space in the midline, the median approach often requires the extraction of a central incisor. This is not only cosmetically bothersome but can create difficulties with closure of the gingival tissue over the extracted socket area and may result in bone exposure and subsequently mal or non-union, bone necrosis or osteoradionecrosis after RT [10]. Advocates of the median approach suggest that this area is usually outside the normal fields of RT hence it is less susceptible to the adverse effects of radiation. In addition, there is unequal muscular pull in the paramedian region, which could lead to nonunion [8]. Finally, the potential damage to the canine in the paramedian approach is detrimental because this tooth is an excellent abutment tooth for prosthetic rehabilitation and is one of the strongest of all the dentition [14].

In the modern literature, there is a preference for the paramedian approach [5,6,9]. A large retrospective review of 220 consecutive patients treated with the paramedian approach found very low complication rates (10.5%) and good esthetic and functional outcomes [9,15]. This study looked at complications related to fixation failure or poor wound healing. Dental complications were not part of the recorded complications. Despite this tendency to advocate for the paramedian approach, no studies have documented an actual advantage. Dai et al. compared the two approaches and found no difference in the rate of complications between the two approaches. They looked at major complications such as ORN and minor complications such as cellulitis. Dental complications were not assessed [8]. Similarly, Nam et al. were unable to demonstrate a meaningful difference between the two approaches in a series of 60 patients [5]. In our study, there were significantly more dental complications in the median group (52% vs. 32%) (p=0.04). We have demonstrated that the interdental space in the midline is significantly less, both at the level of the apex and the crest, thus increasing the risk of root or ligament damage with exposure with the osteotomy. This limited distance in the midline region probably leads to damage to the periodontal ligament during the osteotomy, which could explain the trend for the patients to experience dental sensitivity, as well as a higher rate of periodontal damage. The observation that there is more space in the paramedian region has previously been demonstrated [10,16]. Pan et al. not only demonstrated this interdental relationship but they also showed more angulation between the lateral incisor and canine [16]. They studied 50 Taiwanese patients (mean age: 48 years) and found a mean crestal and apical (mm) distance between the central incisors of 1.70 and 2.75, respectively (as compared to 1.12 and 2.38 in our study). In terms of the distance between the lateral incisor and the canine, it was 2.05 and 3.89, respectively (as compared to 1.32 and 3.25 respectively). However, their study was performed at multiple institutions, which could have lead to possible inter-evaluator differences. One single evaluator (TS) performed all our measurements. Shohat et al. also demonstrated, in a series of young patients (mean age: 25.9 years), a larger distance in the paramedian area [10]. In their study, the mean crestal distance (mm) between the two incisors was 1.51 (as compared to our distance 1.12) and at the apical level 1.88 (2.38). In terms of the distance between the lateral incisor and the canine, it was 1.45 (vs. 1.32 in our study) and 3.94 (vs. 3.25). This observation is clinically important. The fact that there is more space in the paramidline as compared to the midline region is important in maintaining dental integrity during osteotomies. This becomes even more important when you are trying to preserve important teeth such as the canine in patients with a small number of residual teeth. The preservation of such teeth enables the patient to have partial dentures and maintain some level of occlusion in the future.

In terms of plate complications, there were significantly more plate complications in the paramedian group (8.2% vs. 0%) (p=0.04). One factor that may account for this finding is the higher rate of preoperative RT in this group of patients. Due to the limited number of events a MVA was not possible.

This study has limitations. Pre-operative dental assessment was not performed on all the patients. Only 23 of our 118 patients had a pre-operative dental assessment at our institution. Hence pre-operative dental complications could not be clearly assessed. We did not collect data on occlusion and occlusal changes. Due to the retrospective nature of the study, there is likely an underrepresentation of the frequency of dental complications in patients undergoing mandibulotomy. We were not able to evaluate lag screw repair of mandibulotomy sites since this method is not typically used at our institution. The location of the mandibulotomy was based on individual surgeon preference and experience and selection factors may have influenced outcomes in this group of patients. There was a difference in the number of patients treated with pre-operative RT, with higher rates in the paramedian group. This study may be limited in terms of patient numbers. A larger study size would be more desirable. However, given that there are not many large series and no randomized control trials, this study does provide a means to evaluate outcomes as long as we recognize the limitations of retrospective studies.

## Conclusion

Dental complications are relatively uncommon following mandibulotomy. The paramedian osteotomy may have benefits over the median osteotomy in terms of maintaining dentition but may also result in a higher rate of plate complications. There is more interdental space in the paramedian region as compared to the median region. Further investigation is warranted with larger multicenter studies to compare these two approaches.

## Consent

Written informed consent was obtained from the patient for publication of this report and any accompanying images.

## Competing interests

The authors declare that they have no competing interests.

## Authors’ contributions

TS was involved in the study design as well the collection of data, statistical analysis and the drafting and revisions of the manuscript. EB was involved in the study design as well as drafting of the manuscript. HBC was involved in the collection of data as well as the drafting of the manuscript. REW was involved in the study design as well as the drafting and revising the manuscript. EGA was involved in the statistical analysis for the study. DHB was involved in the drafting and revision of the manuscript. RWG was involved in the drafting and revision of the manuscript. PJG was involved in the drafting and revision of the manuscript. JCI was involved in the drafting and revision of the manuscript. JW was involved in the drafting and revision of the manuscript. DPG was involved in the study design, statistical analysis as well as the drafting and revision of the manuscript. All authors read and approved the final manuscript.
